# A skin lesion hair mask dataset with fine-grained annotations

**DOI:** 10.1016/j.dib.2023.109249

**Published:** 2023-05-18

**Authors:** Sk Imran Hossain, Sudipta Singha Roy, Jocelyn De Goër De Herve, Robert E. Mercer, Engelbert Mephu Nguifo

**Affiliations:** a Université Clermont Auvergne, Clermont Auvergne INP, ENSMSE, CNRS, LIMOS, F-63000 Clermont–Ferrand, France; b The University of Western Ontario, Canada; c Université Clermont Auvergne, INRAE, VetAgro Sup, UMR EPIA, 63122 Saint-Genès-Champanelle, France; d Université de Lyon, INRAE, VetAgro Sup, UMR EPIA, F-69280 Marcy l'Etoile, France

**Keywords:** Skin lesion, Hair mask, Hair segmentation, Hair augmentation, Deep learning

## Abstract

Occlusion of skin lesions in dermoscopic images due to hair affects the performance of computer-assisted lesion analysis algorithms. Lesion analysis can benefit from digital hair removal or realistic hair simulation techniques. To assist in that process, we have created the largest publicly available skin lesion hair segmentation mask dataset by carefully annotating 500 dermoscopic images. Compared to the existing datasets, our dataset is free of non-hair artifacts like ruler markers, bubbles, and ink marks. The dataset is also less prone to over and under segmentations because of fine-grained annotations and quality checks from multiple independent annotators. To create the dataset, first, we collected five hundred copyright-free CC0 licensed dermoscopic images covering different hair patterns. Second, we trained a deep learning hair segmentation model on a publicly available weakly annotated dataset. Third, we extracted hair masks for the selected five hundred images using the segmentation model. Finally, we manually corrected all the segmentation errors and verified the annotations by superimposing the annotated masks on top of the dermoscopic images. Multiple annotators were involved in the annotation and verification process to make the annotations as error-free as possible. The prepared dataset will be useful for benchmarking and training hair segmentation algorithms as well as creating realistic hair augmentation systems.

**Specifications Table**

**Table:** 

Subject	Computer Vision and Pattern Recognition.
Specific subject area	Skin lesion hair segmentation, augmentation, and mask generation.
Type of data	Image.
How the data were acquired	Five hundred dermoscopic images covering different hair patterns were collected from ISIC 2018 dataset [1]. The images are CC0 licensed i.e. copyright free and can be redistributed. Segmentation masks were generated using a deep learning model and manual manipulation with Adobe Photoshop software [2].
Data format	Raw, Portable Network Graphics (PNG), Secondary annotated.
Description of data collection	Each sample in the dataset is handpicked to cover a wide range of skin lesion hair types.
Data source location	Primary dermoscopic image source: ISIC 2018 dataset [1] Secondary annotated image source: • Institution: Université Clermont Auvergne^1^, The University of Western Ontario^2^ • City/Town/Region: Clermont Ferrand^1^, London^2^ • Country: France^1^, Canada^2^
Data accessibility	Repository name: Mendeley Data Data identification number: 10.17632/j5ywpd2p27 Direct URL to data: https://data.mendeley.com/datasets/j5ywpd2p27

## Value of the Data

• This is the largest publicly available fine-grained skin lesion hair segmentation mask dataset. High-quality hand-annotated segmentation masks are costly and time-consuming to produce.
• This is the only dataset free of non-hair artifacts.
• This dataset will be useful for proper benchmarking of hair segmentation algorithms, as it is free of non-hair artifacts and segmentation errors.
• Our dataset can be used to train a generative model for automating the task of realistic skin hair mask generation.
• The dataset will contribute to skin lesion research by allowing researchers to train robust skin lesion hair segmentation algorithms.

## 1 Objective

Artificial intelligence-assisted skin lesion analysis is becoming popular nowadays thanks to the advancement in deep learning techniques [3]. However, their performances may be affected by skin hair artifacts [5]. To tackle this issue, researchers are working on digital hair segmentation, removal, and augmentation techniques [4,5]. An accurate hair mask segmentation dataset is needed to properly benchmark the segmentation algorithms. Moreover, existing researches on skin hair augmentation require a hair mask to generate hair in specified locations. These masks are created using pre-segmented hair masks or random lines or curves [4]. A well-annotated hair mask dataset will be effective for training generative models to automate the mask generation process. According to our study, the largest publicly available dataset [5] contains annotations for 306 images but with 18 duplicates and suffers from under-segmentation, and non-hair artifacts. Gallucci [6] created a dataset of 75 images only, which lacks complex patterns and is not a well-representative of the broader skin hair distribution. Akyel et al. [7] prepared a non-public dataset of 2500 images. However, it contains rulers, ink spots, and other noises alongside skin hair. Our motivation for creating the dataset was to resolve the issues in available datasets.

## 2 Data Description

Our dataset is publicly available in a data repository [8]. The dataset contains skin hair annotation masks for 500 dermoscopic images. The dataset is organized into three folders namely *dermoscopic_image, hair_mask*, and *overlay*. Table 1 shows some example images from each of the folders. The *dermoscopic_image* folder contains 500 dermoscopic images handpicked from the primary image source [1] covering different hair patterns. By different hair patterns, we emphasize the variation in terms of width, length, color, shape, and density of skin hairs. We retained the original names of the image files from the primary image source. The *hair_mask* folder contains a binary segmentation mask for each of the images of the *dermoscopic_image* folder. In a segmentation mask image, white pixels represent skin hair and black pixels represent the background. The overlay folder contains hair mask images superimposed on the original dermoscopic images. We provided the superimposed images for easy public verification so that, other people can report any annotation mistakes and contribute to improving the dataset. Images in the *hair_mask* and *overlay* folders share the same names as the primary images in the *dermoscopic_image* folder.

**Table 1:** Details of dataset with examples.

## 3 Experimental Design, Materials and Methods

Annotating skin lesion hair from scratch is a tedious task. To ease the process, we trained a U-Net [9] deep segmentation model using a weakly annotated dataset provided by Li et al. [5]. The codes used for the process are available in the *unet* folder under the *additional_materials* folder of our data repository [8]. Inside the *unet* folder the U-net model is defined in *model.py* file, unet training is performed using the *unet_training.ipynb* python notebook file, and the task of predicting initial masks for the dermoscopic images are done using the *predict_mask.ipynb* file.

Using the trained U-net we extracted the initial hair mask for 500 handpicked copyright-free dermoscopic skin lesion images from ISIC 2018 dataset [1] to cover different hair patterns. The resulting masks suffer from various segmentation errors like under-segmentation, over-segmentation, and non-hair artifacts. We involved three independent annotators for the correction of the segmentation errors.

The first annotator manually corrected all the found segmentation errors with Adobe Photoshop software [2]. A video demonstration of the segmentation mask editing process using Adobe Photoshop software is available in the *mask_editing_process.mp4* file of the *additional_materials* folder of our data repository [8]. The steps involved are as follows:

1. Open the dermoscopic image in Adobe Photoshop.
2. Open the initial segmentation mask image in Adobe Photoshop and copy it on top of the dermoscopic image.
3. Change the blending mode of the mask image to “*screen”*.
4. Select the brush type as hard brush (hardness of the brush set to 100 percent).
5. Remove unwanted segmentation marks from the mask image by painting with a black brush.
6. Adjust the brush size according to the width of the skin hair and add missing segmentation marks to the mask image by painting with a white brush.
7. Change back the blending mode of the mask image to “*normal”*.
8. Make additional adjustments to the segmentation mask if required.
9. Save the finalized segmentation mask image in the desired format.

**Fig 1:**
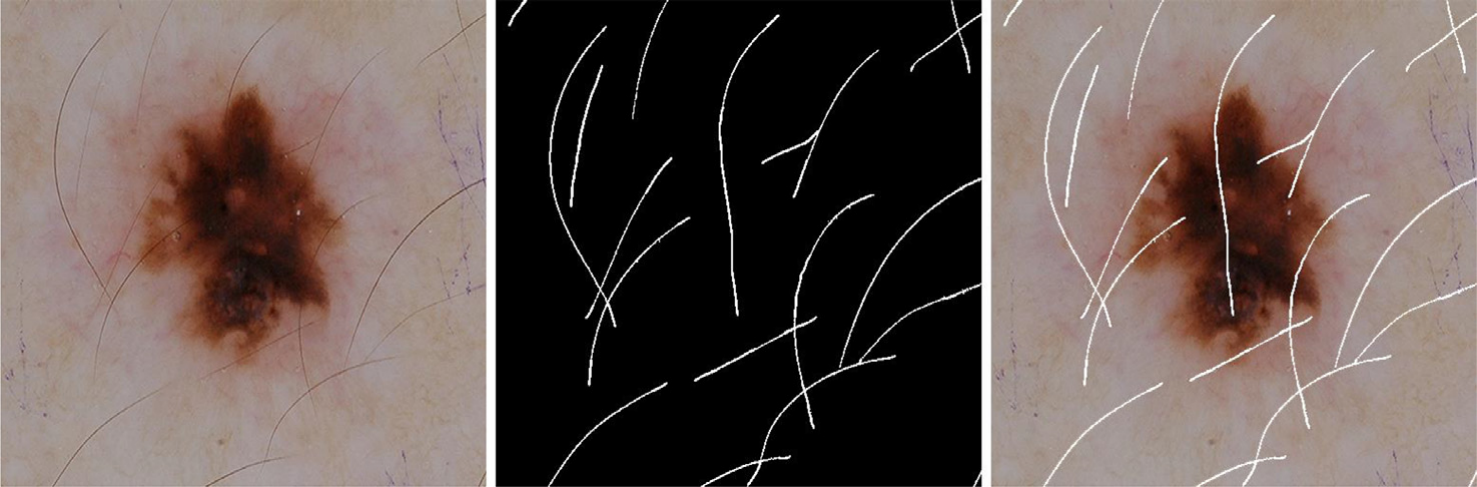
Image collage for easy verification.

**Fig 2:**
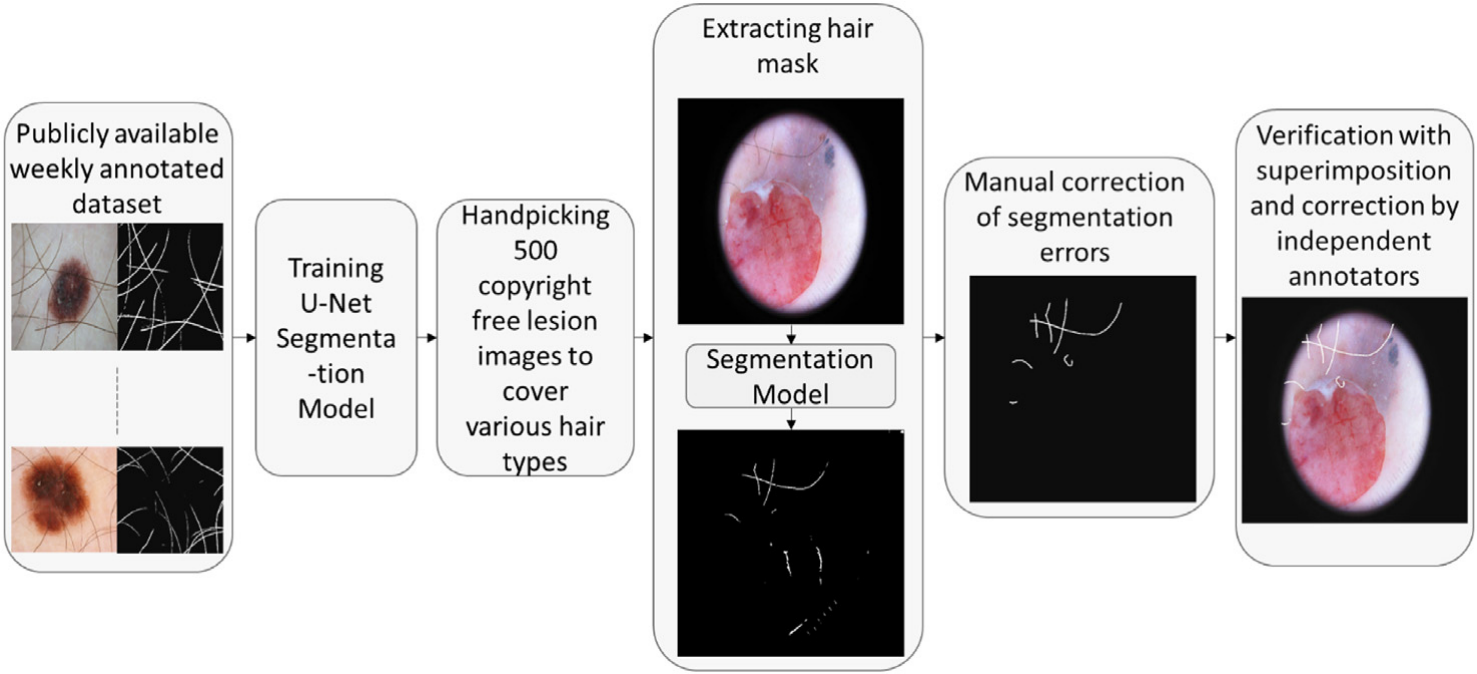
Dataset creation workflow.

To verify the quality of the annotation first we binarized each corrected mask to make sure every pixel is either black or white. Then, we made the black pixels of the mask image fully transparent and superimposed it on the original dermoscopic image. Finally, we created a collage of three types of images: dermoscopic image, corrected mask, and the superimposed image as shown in Fig. 1 for easy verification. The codes used for these operations are available in the *check_annotation.ipynb* file of the *additional_materials* folder of our data repository [8]. Using the image collage a second annotator marked errors missed by the first annotator. A third annotator corrected the mistakes identified by the second annotator, which was finally reverified by the first annotator. We tried to make the annotations as error-free as possible. The overall dataset creation workflow is shown in Fig. 2.

## Ethics Statements

Not applicable.

## CRediT authorship contribution statement

**Sk Imran Hossain:** Conceptualization, Methodology, Software, Data curation, Writing – original draft, Visualization. **Sudipta Singha Roy:** Data curation, Writing – original draft. **Jocelyn De Goër De Herve:** Resources, Writing – review & editing. **Robert E. Mercer:** Data curation, Writing – review & editing. **Engelbert Mephu Nguifo:** Writing – review & editing, Supervision, Project administration, Funding acquisition.

## Declaration of Competing Interest

The authors declare that they have no known competing financial interests or personal relationships that could have appeared to influence the work reported in this paper.

## Data Availability

A skin lesion hair mask dataset with fine-grained annotations (Original data) (Mendeley Data).
